# The complete mitochondrial genome of *Acanthopagrus schlegelii* (Perciformes: *Sparidae*) with phylogenetic consideration

**DOI:** 10.1080/23802359.2016.1144105

**Published:** 2016-04-06

**Authors:** Xilan Ma, Zhenzhen Xie, Libin Zhou, Yongzhi Chen

**Affiliations:** a Huizhou University, Huizhou, China;; b State Key Laboratory of Biocontrol, Institute of Aquatic Economic Animals, and the Guangdong Province Key Laboratory for Aquatic Economic Animals, Sun Yat-Sen University, Guangzhou, China

**Keywords:** *Acanthopagrus schlegelii*, mitochondrial genome, phylogenetic

## Abstract

The complete mitochondrial genome of the *Acanthopagrus schlegelii* was presented in this study. The mitochondrial genome is 16 798 bp long and consists of 13 protein-coding genes, two rRNA genes, 22 tRNA genes and a control region. The gene order and composition of *Acanthopagrus schlegelii* mitochondrial genome was similar to that of most other vertebrates. The nucleotide compositions of the light strand in descending order is 28.04% of T, 27.94% of G, 27.87% of A and 16.15% of C. With the exception of the NADH dehydrogenase subunit 6 (ND6) and eight tRNA genes, all other mitochondrial genes are encoded on the heavy strand. The phylogenetic analysis by maximum-likelihood (ML) method shown that the *Acanthopagrus schlegelii* has the closer relationship to the *Rhabdosargus sarba* in the phylogenetic relationship.

The *Acanthopagrus schlegelii* is a commercially important aquaculture fish species, which mainly distributes in the Northwest Pacific region, including southern Hokkaido, Japan to the southern Korean Peninsula, middle and north coast of the Republic of China and Taiwan. With the aim of achieving to find new DNA markers for the studies on population genetics of *A. schlegelii*, we determined to sequence the complete mitochondrial genome of *A. schlegelii* using the next-generation sequencing (NGS) techniques strategy (Xie et al. 2015). The specimen was obtained from the Daya Bay Aquaculture Center, Guangdong, China. Then the specimen was preserved in 95% ethanol. The total genomic DNA was extracted from the fin of the fresh fish using the salting-out procedure (Howe et al. 1997).

The complete mitochondrial genome of *A. schlegelii* (Genbank accession no. KT805958) is 16 798 bp in length, consisting of 13 protein-coding genes, two ribosomal RNA genes (12S rRNA and 16S rRNA), 22 transfer RNA genes (tRNA) and one control region, which is the same as the typical vertebrates (Wang et al. 2008). Most of the genes are encoded on the heavy strand, with only the NADH dehydrogenase subunit 6 (ND6) and eight tRNA genes [*Gln, Ala, Asn, Cys, Try, Glu, Pro* and *Ser* (GCT)] encoded on the light strand. Overall nucleotide compositions of the light strand are 27.87% of A, 16.15% of C, 28.04% of T and 27.94% of G. However, the most representative base is T and the bias against C was observed, which is different from the base compositions of mitochondrial genome of other teleosts.

All the protein-coding genes begin with an ATG start codon except for COX1 started with GTG. Five types of stop codons revealed are TAA (*ND4L, ND5, ND1,* and *ND6*), TA (*ND2, ATP6,* and *COXIII*), T (*COXII, ND3, ND4,* and *CYTB*), AGG (*COXI*) and TAG (*ATP8*). The 12S and 16S rRNA genes are located between the *tRNA-Phe* (GAA) and *tRNA-Leu* (TAA) genes, and are separated by the *tRNA-Val* gene with the same situation found in other vertebrates. Most genes are either abutted or overlapped. The 22 tRNA genes vary from 67 to 73 bp in length. All these could be folded into the typical cloverleaf secondary structure although numerous non-complementary and T–G base pairs exist in the stem regions. The control region was 1098 bp in length, located between *tRNA-Pro* (TGG) and *tRNA-Phe* (GAA) gene. The nucleotide composition of control region was 33.33% of A, 21.73% of C, 13.92% of G and 31.01% of T.

The phylogenetic position of *Acanthopagrus schlegelii* was reconstructed with the complete mtDNA sequences from 14 species of Perciformes by using the maximum-likelihood (ML) methods (Kumar et al. 2004). As shown in Figure 1, the *Acanthopagrus schlegelii* has the closer relationship to the *Rhabdosargus sarba*.

**Figure 1.**
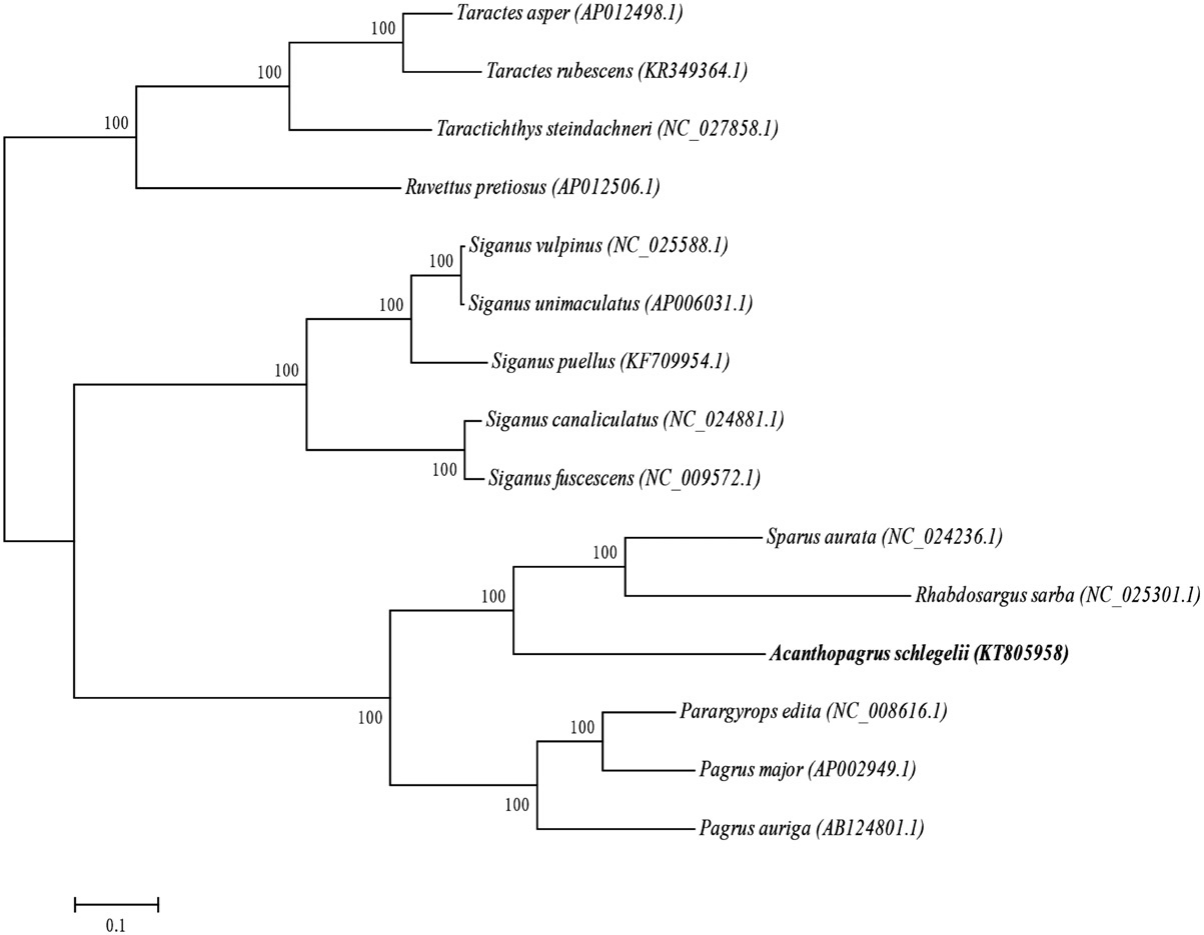
The ML phylogenetic tree of Perciformes species. Numbers on each node are bootstrap values of 100 replicates.

Furthermore, the *Rhabdosargus sarba* and *Sparus aurata* clustered into a monophyletic group, which suggested the *Acanthopagrus schlegelii* also has the closer relationship to the *Sparus aurata*.
